# Revisiting Eck and Dayhoff’s Building Block Model of Ferredoxin Evolution on Dayhoff’s 100th Birthday

**DOI:** 10.1007/s00239-025-10283-3

**Published:** 2025-11-06

**Authors:** Gustavo Caetano-Anollés

**Affiliations:** Evolutionary Bioinformatics Laboratory, Department of Crop Sciences and Carl R. Woese Institute for Genomic Biology, University of Illinois at Urbana-Champaign, Urbana, IL 61801 USA

**Keywords:** Bioinformatics, Folds, Loops, Molecular evolution, Origin of life, Phylogenomic analysis, Prior molecular states, Structural domains

## Abstract

Nearly 60 years ago, Eck and Dayhoff (Science 152:363–366, 1966) aligned amino acids from first and second halves of a ferredoxin sequence, revealing a symmetric CX_2_CX_2_CX_3_CX_18_CX_2_C_2_C_3_C spacing signature in which X_n_ denoted intervening residues. This symmetry, along with other cyclic patterns, suggested that a tandem duplication shaped ferredoxin evolution and that the ancestral sequence may have drawn from a reduced amino acid repertoire. Here, I revisit the duplication model using the deep learning-based AlphaFold2 ab initio pipeline, benchmarked against the I-TASSER threading tool. Predicted ancestral structures were obtained with high confidence, with some aligning to the two halves of a reference ferredoxin (PDB entry 1CIF) at acceptable RMSD and TM-score values. A chronology of loops and structural domains further identified which duplicate was ancestral, reinforcing the antiquity of the fold. Loops and domains also dissected the evolution of the [4Fe–4S] ferredoxin superfamily. The resulting structural models provided strong support for the tandem duplication hypothesis and the idea that modular units underpinned early molecular evolution. However, they also challenged the notion that the duplication event arose from a reduced amino acid alphabet. This work revisits Eck and Dayhoff’s seminal insights and commemorates Dayhoff’s pioneering contributions on the centenary of her birth.

**Supplementary Information:**

The online version contains supplementary material available at 10.1007/s00239-025-10283-3.

## Introduction

Nearly 60 years ago, Richard Eck and Margaret Dayhoff proposed the existence of building blocks in biochemical evolution (Eck and Dayhoff 1966). They used a ferredoxin sequence that had been recently acquired from *Clostridium pasteurianum* (Tanaka et al. 1964) to explore the origin of the molecule. Ferredoxins are iron-sulfur (Fe–S) proteins that mediate electron transfer in metabolic reactions (Meyer 2008). The ferredoxin that was used belongs to a large group of small molecules known as low-potential [4Fe–4S] ferredoxins, which hold one or two tetrahedral iron-sulfur clusters, each bound to four conserved cysteine residues and are widely distributed in Bacteria but also in the other superkingdoms (Nzuza et al. 2021). Iron-sulfur clusters are believed to be crucially linked to prebiotic chemistry and a pyrite-pulled chemoautotrophic origin of life (Wächtershäuser 1992). Clusters often form spontaneously in solution and could have easily formed in prebiotic reactions at the surface of growing pyrite crystals. They probably had catalytic activities even in the absence of proteins (Russell and Martin 2004). Emerging polypeptide enzymes with redox and non-redox functions may have taken advantage of these primordial iron-sulfur cluster chemistries, especially enzymes harboring fold structures of iron-nitrogenase and iron-hydrogenase pioneers that specialized by suppressing hydrogen or nitrogen production, respectively.

Ferredoxin clusters are embedded in a α + β sandwich fold with antiparallel β-sheet in an [α-β-α]_2_ conformation with pseudo-C2 symmetry (Meyer 2008). The fold is old and widely distributed. It belongs to a large class of Fe-S protein folds that coordinate iron atoms predominantly via cysteinyl sulfur residues and likely originated alongside the emergence of the first cysteine biosynthetic pathway (Zhang et al. 2012). Eck and Dayhoff investigated the origin of ferredoxin, reasoning that Fe–S proteins represented some of the early catalysts in biochemical reactions. They aligned amino acids of the first half of the ferredoxin molecule to those of the second half revealing a symmetric CX_2_CX_2_CX_3_CX_18_CX_2_C_2_C_3_C spacing signature (subtype 9 of 2[4Fe-4S] ferredoxins; Nzuza et al. 2021), with X_n_ denoting intervening residues. The symmetry, together with other repeating patterns, suggested that ferredoxins arose through tandem duplications from a sequence with a reduced amino acid repertoire.

Here, I revisit this duplication model by modeling the proposed ancient duplication with the deep learning-based Alphafold2 pipeline that derives atomic structures directly from amino acid sequences at experimental resolution. I test whether the sequence-level predictions made by Eck and Dayhoff are borne out at the structural level and integrate these results with timelines of protein domains and loop prototypes to estimate the antiquity of the duplication event that gave rise to the ferredoxin fold. The study highlights Eck and Dayhoff’s seminal insight into protein evolution while commemorating Dayhoff’s pioneering contributions on the centenary of her birth.

## Materials and Methods

Atomic structures were predicted ab initio with the AlphaFold2 pipeline (Jumper et al. 2021) using a local ColabFold implementation of the software (Mirdita et al. 2022). Multiple sequence alignments (MSAs) were built using MMseqs2-based homology searches against UniRef100, PDB70 and an environmental sequence set, which were used for 3 recycling rounds of structure inference. Accuracy was measured with the predicted local distance difference test (pLDDT), which provides a per-residue estimate of prediction confidence, and the predicted aligned error (PAE), which measures confidence in the relative positions of pairs of residues. pLDDT ‘confidence bands’ of expected prediction reliability are the following: >90, models with very high confidence; 90 − 70, models with confidence, showing good backbone predictions; 70 − 50, models with low confidence; and < 50, models with very low confidence, generally showing ribbon- like structures. pLDDT < 60 can be considered a reasonably strong predictor of intrinsic disorder. PAE evaluates the cohesiveness of structural modules. AlphaFold2 models and associated data can be downloaded from Model Archive (Tauriello et al. 2025) under global accession code ma-gca-fd (doi: 10.5452/ma-gca-fd). Data includes PDB-formatted model files with local per-residue model confidence scores (pLDDT) for 5 ranked structures sorted by average pLDDT, PAE and corresponding score files containing a PAE array and a list with the average pLDDT and pTM score for best structure, PAE plots measuring confidence in the relative position of pairs of residues, plot of MSA coverage, plots of per-residue pLDDTs, and A3M-formatted input MSA.

AlphaFold2 models were benchmarked against models generated by threading with I-TASSER (Yang and Zhang 2015; Zheng et al. 2021). I-TASSER selects structure templates generated by the LOMETS meta-server threading approach that are of highest Z-score significance and uses them in simulations to generate many structural conformations, which are then clustered based on pair-wise structural similarity and used to define the best 5 models.

Structural alignments and visualizations were conducted using Chimera (Pettersen et al. 2004). Reference experimental structures and ab initio models were superimposed using the MatchMaker and MatchAlign tools to identify structural divergences. Topological similarities were evaluated with average template modeling scores (TM scores) using US-align (Zhang et al. 2022). TM-scores lower than 0.30 indicate random structural similarity and those higher than 0.5 indicate identical folds.

The times of origin of Fe-S protein domains defined at family level of the Structural Classification of Proteins (SCOP) (Murzin et al. 1995) were obtained from a published chronology of domains that was reconstructed from a phylogenomic analysis of 8127 reference-quality proteomes of cellular organisms and viruses (Mughal et al. 2020). Times of origin were provided as *node distances* (*nd*) in a relative scale. A molecular clock of folds allowed to report times of origin in a scale of billions of years (Gy) (Wang et al. 2011). Domain families (leaves of the tree) were identified with SCOP concise classification strings (*ccs*). For example, the short chain ferredoxin family of SCOP is labeled d.58.1.1, with d representing the protein class (alpha and beta proteins), 58 representing the fold (ferredoxin-like), 1 the superfamily (4Fe–4 S ferredoxins) and 1 the family (short-chain ferredoxins). Protein loop prototypes defined by the ArchDB repository (Bonet et al. 2014) were mapped onto domain chronologies to trace evolutionary recruitment using previously generated evolving networks (Aziz et al. 2023). In these networks, the time of origin of domains was transferred directly to loop prototypes assuming the age of the loop is the age when the loop function is first transferred between structural scaffolds. Prototypes were identified using a Density Search (DS) algorithm, which locates areas in the feature space where loops cluster densely around a centroid characterized by their length, conformation, and geometric properties.

## Results and Discussion

### Retrodictive and Predictive Pathways

**Fig. 1:**
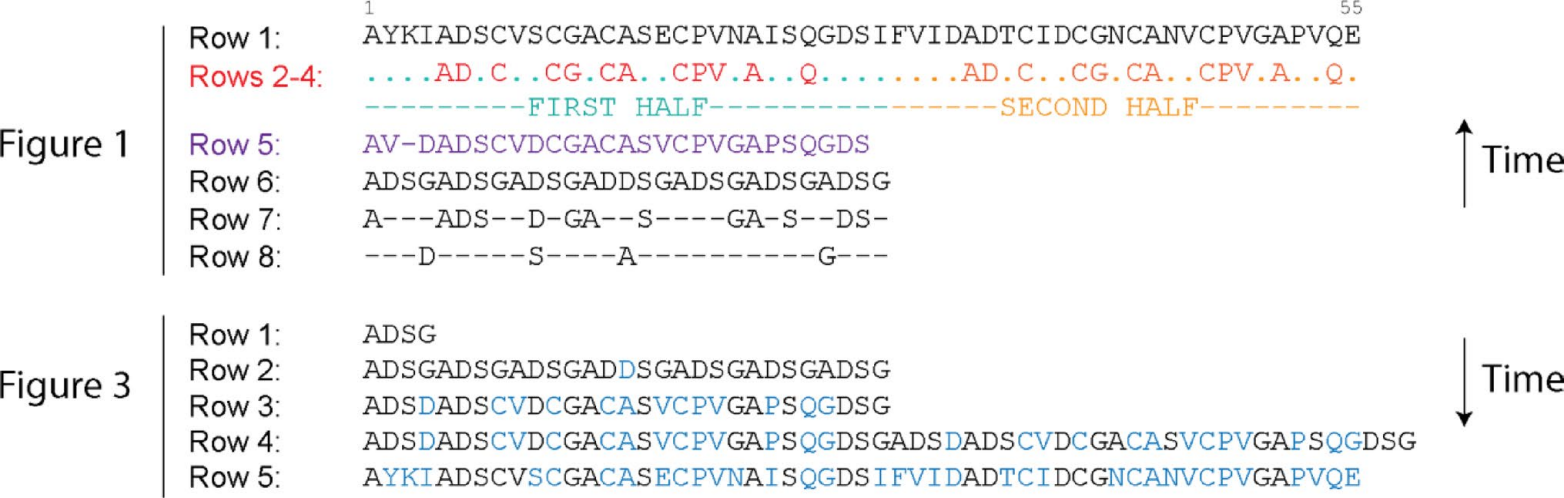
Eck and Dayhoff’s amino acid alignment against a ferredoxin sequence from *Clostridium pasteurianum* (row 1). In their Figure 1 , they revealed a repeating pattern of amino acids (repeated motif distilled in rows 2–4), which define a first and second half of the molecule (colored red and orange) and an ancestral sequence in purple (row 5) containing the 7 amino acids found in the repeated motif plus serine (S) residues, whenever they occurred in either half. Rows 6–8 reveal cyclic patterns that are used to reduce the amino acid alphabet to only four amino acids (A, D, S and G). Time flows backwards in this (retrodictive) alignment scheme. In their Figure 3, they regenerated the modern ferredoxin sequence from an ancestral ADSG motif by a series of duplications and mutations (residues labeled in blue). Time flows forward in this (predictive) alignment scheme

The landmark bioinformatic study of Eck and Dayhoff (1966) was among the first to apply amino acid sequence alignment and to make informal uses of the one-letter amino acid code of proteins. In their Fig. 1, they identified a symmetric CX_2_CX_2_CX_3_CX_18_CX_2_C_2_C_3_C spacing signature, along with other cyclic patterns, through an analysis of eight rows of amino acid sequences, some of which they aligned. Row 1 presented the 55 amino acid residues of the ferredoxin sequence. The two halves of the molecule were then aligned to each other (rows 2 and 3), revealing 12 residue coincidences (row 4), a number significantly greater than expected by chance (*p* < < 0.001). From the 7 amino acids that were present in both halves, plus S when it occurred in either half, they reconstructed a presumed ancestral sequence (row 5). This sequence showed C residues recurring in cycles of three and A residues in cycles of four, with a discontinuity at midpoint position 15. From the cyclic patterns, they derived a repeating four-amino-acid motif (ADSG; row 6), which they then used to reduce the 27-residue sequence of row 5 into a 13-residue sequence that conformed to row 6 (row 7) and to a sequence of residues that did not (row 8). This analysis revealed the symmetric spacing signature of ferredoxin. It also provided rules to invert the back-in-time process of Fig. 1 and build a scenario of origin and evolution of the molecule, which they described in their Fig. 3. In this reconstruction, they doubled the ADSG repeat (row 1) seven times (row 2), incorporated the effects of mutation (row 3), and a putative duplication caused by ‘chromosomal ‘aberration’ (row 4), ultimately arriving at the sequence of the extant molecule by accounting for residues altered during evolution (row 5). Eck and Dayhoff’s approach thus combined retrodictive (back-in-time) and predictive (into-the-future) modeling (Fig. 1).

### Testing the Duplication Model with *ab initio* Structural Predictions

**Fig. 2:**
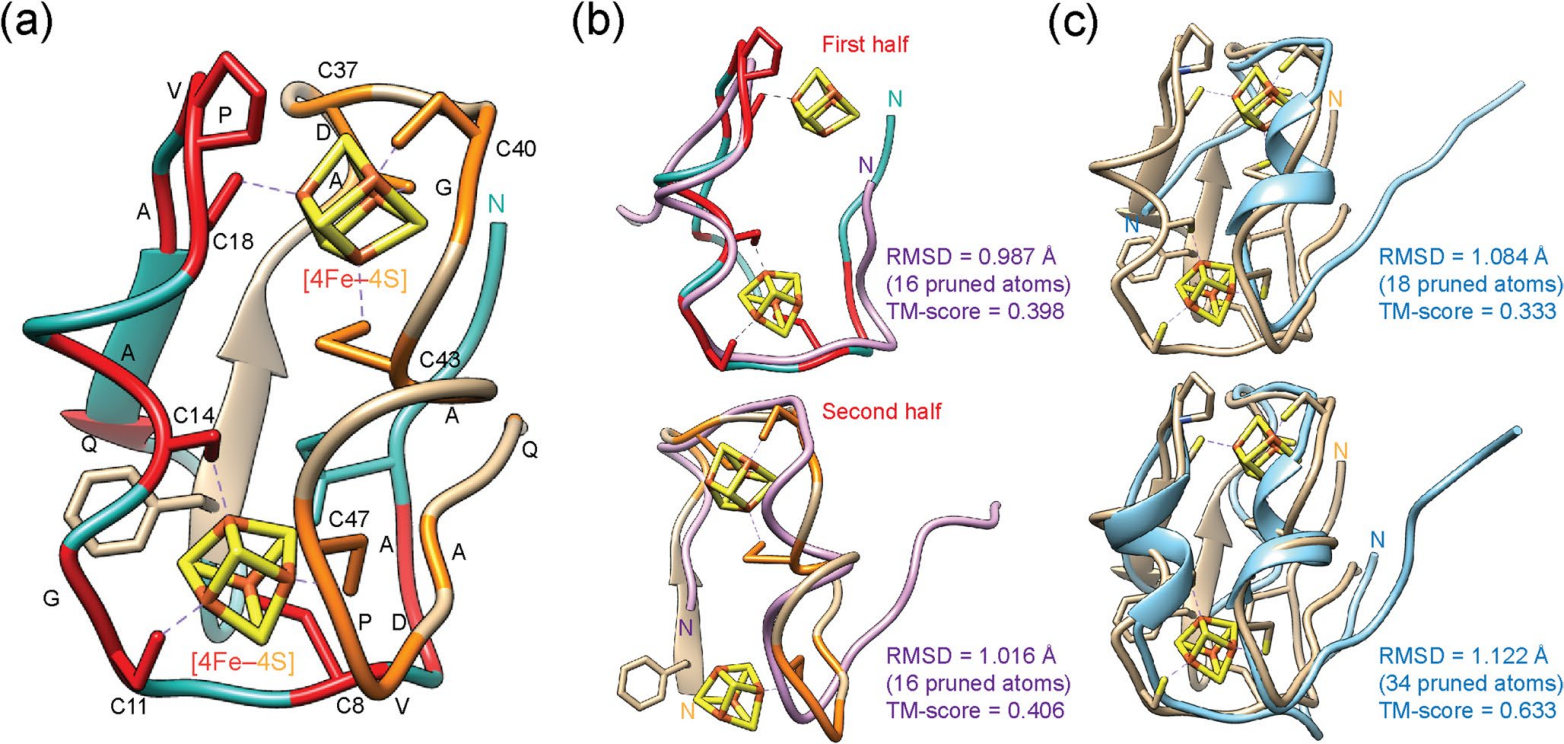
Checking the validity of the tandem duplication model of Eck and Dayhoff with a solution NMR structural model of ferredoxin and AlphaFold2 structural predictions of ancestral molecules. **a** A model of the *Clostridium pasteurianum* ferredoxin (1CLF, conformer 1) from a family of atomic structures acquired by ^1^H-NMR (Bertini et al. 1995) was colored according to the proposed two halves (light sea green and tan) highlighting conserved amino acid signatures (red and orange) that make up the [4Fe–4S]-binding sites. **b** An AlphaFold2 structural model of the ‘retrodicted’ ancestral sequence of row 5 of Eck and Dayhoff’s Fig. 1 (purple) was aligned against each of the two halves. **c** AlphaFold2 structural models of the ‘predicted’ ancestral sequence of row 3 of Fig. 3 (blue), which precedes the major duplication (top image), and the ‘predicted’ ancestral sequence of row 4 of Fig. 3 (blue), which follows the duplication (bottom image), were aligned against the experimental model of ferredoxin. Median backbone accuracy RMSD and residue coverage (Cα atoms) together with TM-score are given for each structural alignment. The N-terminal residues are indicated in all structural models

To test and validate their duplication model, I first mapped the two halves and their conserved amino acids to atomic structures acquired by solution ^1^H-NMR (Bertini et al. 1995)(Fig. 2a). I then used the state-of-the-art deep learning AlphaFold2 ab initio pipeline to predict the structure of the ‘retrodicted’ ancestral sequence that gave rise to the duplication (row 5 of their Fig. 1). The structural model was obtained with high confidence (global model confidence score, pLDDT = 78.5) and aligned well to the two halves of the molecule (Fig. 2b). Alignments against the first and second halves were reasonable, especially around regions holding the active sites, and recovered correct global topologies (TM-scores of 0.398 and 0.406, respectively), providing atomic-level support for the tandem duplication model and the existence of evolutionary building blocks. I also modeled the structure of the ‘predicted’ ancestral sequences that preceded and followed the putative duplication (rows 3 and 4 of their Fig. 3). These models were of low confidence (pLDDT = 69.6 and 65.2, respectively) but nonetheless aligned well with the extant ferredoxin sequence and recovered correct global topologies (TM-scores of 0.333 and 0.633, respectively) (Fig. 2c). The three AlphaFold2 models were compared against top models generated by threading with I-TASSER (Supplementary Fig. 1). This benchmarking enabled identification of known structures, ligands and ligand sites in extant proteins that agreed with the predictions. Supplementary Table 1 lists top threading templates from the LOMETS threading programs for each predicted structure, with the 2[4Fe–4S] ferredoxins of *Peptostreptococcus asaccharolyticus* (1DUR_A) and *C. pasteurianum* (1CLF_A) frequently ranking among the best matches. Predicted ligand binding sites accommodated [3Fe–4S] and [4Fe–4S] clusters with tetrahedral coordination, consistent with those found in low potential 2[4Fe–4S] ferredoxin molecules.

In contrast, AlphaFold2 modeling of the deeper ancestral building blocks made of cycles of ADSG subsequences separated by a discontinuity in position 15 (Eck and Dayhoff’s row 6 of Fig. 1, which is identical to row 2 of their Fig. 3) produced unstructured coils (Supplementary Fig. 2a) with low confidence (pLDDT = 66) that failed to be aligned to the two halves of the molecule. These results make deeper evolutionary inferences of a reduced amino acid repertoire and associated assumptions less likely. Threading with I-TASSER returned top ranking structures associated with amyloid β–peptides (1BA6_A), a pentapeptide repeat protein from *Nostoc* filamentous cyanobacteria (6OMX_A), or synthetic β–solenoid proteins (4YFO_A) (Supplementary Table 1), none of which interacted with ligand Fe-S clusters. The best-threaded structural model aligned poorly–only 5 out of 27 residues matched–with a TM-score of 0.192 to the ancestral sequence that gave rise to the duplication (row 5 of Fig. 1). Its predicted binding sites accommodated a complex diphosphate molecule (TP9) that interacts with thiamin diphosphate-dependent enzymes (2IHU_B) (Supplementary Fig. 2b) or interfaced with a synthetic 2-phenylamino-4-methyl-5-acetylthiazole inhibitor of a bacterial fatty acid synthase (2VBA_O).

AlphaFold2 uses neural networks to predict inter-residue distance and orientation distributions directly from MSAs of homologous sequences. The tool however can also produce accurate models for novel folds in absence of homologous templates. To test if other methods that do not rely so heavily on sequence homology would fold the predicted ADSG-rich molecule, I explored the template-free protein structure prediction pipeline DeepFold that focuses on spatial restraint potentials and statistical energy functions (Pearce et al. 2022) and the hybrid C-QUARK method that integrates deep-learning-predicted contact maps with traditional fragment-based assembly simulations (Mortuza et al. 2021). As with AlphaFold2, both methodologies produced coils with low confidence that could not be appropriately aligned to the two halves of the ferredoxin molecule (data not shown). This strengthens the conclusion that ADSG-rich ancestors were unlikely.

While proteins were likely made of a few amino acids during the early development of the protein biosynthetic machinery and the ‘standard’ genetic code 3 Gy ago (Wang et al. 2025), the origin of the 2[4Fe–4S] ferredoxins is relatively recent compared to other Fe–S proteins (Zhang et al. 2012) and may have benefitted from the cooption of loops or other modular structures from older Fe–S proteins. This could explain why the repeating ADSG motif does not fold into a functional structure. Note however that the electron transfer functions of the 2[4Fe–4S] ferredoxins do not require the complete ferredoxin fold. In fact, 16 residue-long apo-peptides (maquettes) have been designed that bind to [4Fe–4S] clusters, act as electron acceptors during light-initiated electron transfer, assemble into tetra α-helix bundles, and bind irreversibly to photosystem I complexes (Gibney et al. 1996; Antonkine et al. 2009). Moreover, synthetic symmetric ferredoxins have been constructed that are capable of binding two [4Fe–4S] clusters and support electron transfer in vivo in *Escherichia coli* (Mutter et al. 2019). This modularity suggests that early symmetric ancestral proteins could have been assembled piecemeal from rudimentary building blocks.

Overall, the structure modeling results support the occurrence of a tandem duplication during ferredoxin evolution, but casts doubt on the hypothesis that a reduced amino acid repertoire drove the duplication event.

### Exploring the Origin of the Tandem Duplication

**Fig. 3:**
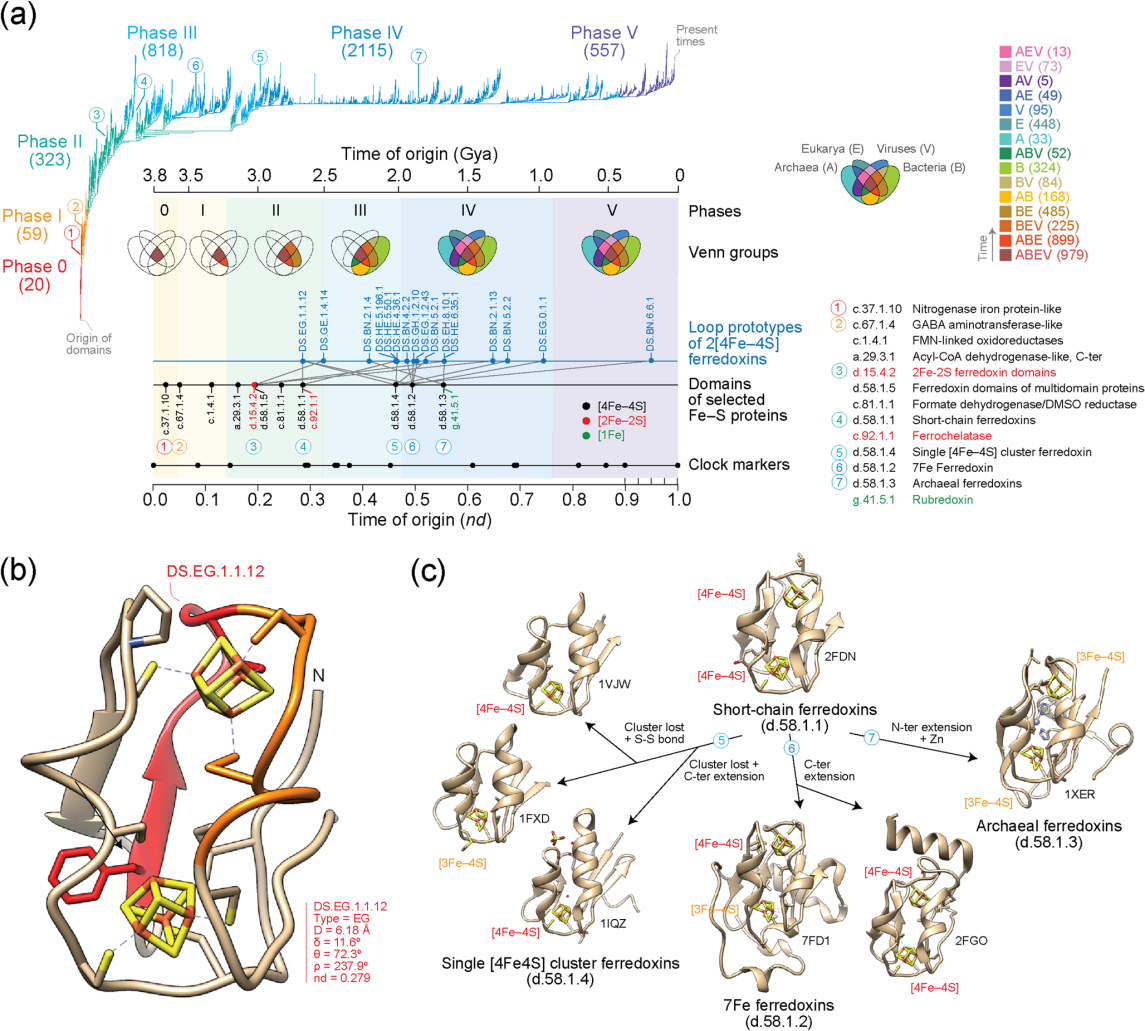
Tracing the evolution of Fe-S proteins and 2[4Fe–4S] ferredoxins. **a** A phylogenomic tree of structural domains defined at fold family level of SCOP ((2,083,556 steps; retention index = 0.704; g1 = 0.0004; Mughal et al. 2020) describes the evolution of 3892 domains, which can be visualized with a timeline of domains (inset). Time of origin was expressed as node distance (*nd*) or as billions of years (Gy) ago. Domains were identified with SCOP concise classification strings (*ccs*). Venn diagrams describe the distribution of families among superkingdoms and viruses and dissect the timeline into 6 evolutionary phases, which are mapped by coloring to the branches of the tree (numbers in parentheses describe new families appearing in evolution). A timeline of protein loop prototypes of the [4Fe–4S] ferredoxin superfamily (d.58.1) mapped to corresponding domain families show extensive episodes of recruitment. **b** An ancient loop prototype is embedded in the ferredoxin structure of short-chain ferredoxins (d.58.1.1). The *Clostridium pasteurianum* ferredoxin model (1CLF, conformer 1) shows correspondences to the MCL.EH.9.3.1 loop prototype, which is colored in red and orange, and to the oldest loop prototype of the molecule (the DS.EG.1.1.12), which is colored in red. **c** The evolutionary timeline of the 2[4Fe–4S] ferredoxins supports the model proposed by Meyer (2008) but dissects the gradual origin of families from ancestral short-chain ferredoxins by loss of clusters and extensions of the molecular termini

Molecular chronologies and time-varying networks built with modern methods of phylogeny reconstruction allow to study the history of biological systems and subsystems and the rise of biological innovations (Caetano-Anollés et al. 2021). Since the antiquity of the ferredoxin fold structure and the origin of the tandem duplication remain important questions, I used chronologies of structural domains and protein loops to explore the time of origin of Fe–S proteins and the 2[4Fe–4S] ferredoxins and their variants (Fig. 3). Domain chronologies were directly derived from a published phylogeny of domains defined at SCOP fold family level (Mughal et al. 2020). Because the rooted optimal tree was significantly imbalanced, times of origins were measured in a relative scale of node distance (*nd*) from the root to the leaves of the tree measuring relative ancestry in a scale from 0 (origin of proteins) to 1 (the present). A clock of folds (Wang et al. 2011) calibrated with biomarkers and geomarkers in Gy linked domain evolution and the geological record (Caetano-Anollés et al. 2021). Protein loops defined by geometry and conformation were sourced from ArchDB (Bonet et al. 2014) and traced along the timelines of domains to reveal networks of loop recruitment (Aziz et al. 2023). Since protein loops serve as precursors to domain structures, investigating their evolution alongside that of domains provides insight into how these two interconnected ancestral molecular forms arose in the protein world (Caetano-Anollés et al. 2024).

Previous studies showed that Fe–S proteins originated quite early in protein evolution (Zhang et al. 2012) and were likely present in the last universal common ancestor (LUCA) of cellular life (Weiss et al. 2016). A chronology of SCOP domains revealed that the first folds and fold superfamilies of Fe–S proteins were associated with the nitrogenase iron protein and GABA aminotransferase structures, which appeared 3.5 billion years (Gy) ago together with the tRNA-dependent cysteine biosynthetic pathway (Zhang et al. 2012). Moreover, an iterative phylogenomic reconstruction of the minimum and maximum structural domain sets of LUCA showed that the SCOP fold superfamilies of those early proteins (SCOP c.37.1 and c.67.1) belonged to the minimum urancestral set of 70 superfamilies that was likely present 2.9 Gya (Kim and Caetano-Anollés 2011) during the start of planet oxygenation (Kim et al. 2012). Consistent with these results, the chronology of domains that I here examine showed that the first Fe–S domain families, the [4Fe–4S]-containing nitrogenase iron protein-like (c.37.1.10) and the [2Fe–2S]-containing GABA aminotransferase-like (c.67.1.4) families, appeared at the base of the tree and very early in the timeline (Fig. 3a). They had times of origin (*nd*) of 0.036 and 0.052, respectively. The molecular clock of folds (Wang et al. 2011) placed the origin of these first Fe-S domains 3.7–3.6 Gy ago. The distribution of SCOP families across Archaea, Bacteria, Eukarya, and viruses represented as a Venn diagram with Venn groups capturing sharing patterns among these supergroups, showed these two early fold families were universal (they belonged to the ABEV Venn group). They were found in 99–100% of cellular proteomes and in 0.05% of viruses examined. A specific temporal sequence of Venn-group emergence, which consistently recovers six evolutionary phases (*see* Caetano-Anollés et al. 2024), revealed that the nitrogenase iron protein-like family appeared in Phase 0 (nulla; the communal world), which includes only universal families (Fig. 3a). In turn, the GABA aminotransferase-like family appeared later, in Phase I (rise of viral ancestors), which defines an ancestral stem line of cellular descent distinct from that of viruses and includes both universal families and few families shared across cellular superkingdoms (ABEV and ABE).

The origin and evolution of nitrogen fixation in our planet is enigmatic yet central to our understanding of biogeochemical cycles (Rucker and Kaçar 2024). Nitrogen fixation links the cycling of metals and nitrogen with the emergence of the nitrogenase enzyme complex. Isotopic evidence from Archaean sediments indicates that a nitrogenase-like ancestor was already present 3.2 Gy ago in environments rich in bioavailable iron, a key component of the nitrogenase complex (Stüeken et al. 2015). Modern research suggests the more ancient molybdenum-dependent nitrogenase, a two-component metalloenzyme, arose from a ‘maturase-like’ non-nitrogen fixing precursor linked to the cofactor maturase (NifEN) of the nitrogenase complex (Garcia et al. 2022). The nitrogenase consists of a [Fe4–S4] cluster-containing homodimeric iron protein (NifH reductase), which transfers electrons, and a heterotetrameric molybdenum-iron protein (NifDK nitrogenase), which reduces nitrogen. Notably, cofactor maturases (nifEN) can reduce nitrogen at the surface of the L-cluster ([Fe_8_S_9_C]), a structural and functional homolog of the M-cluster ([MoFe_7_S_9_C]) of NifDK (Lee et al. 2024). This strengthens the hypothesis of a maturase-like origin of the nitrogenase ancestor.

The early appearance of the nitrogenase iron protein-like family (c.37.1.10) in the Archean, which includes NifH, suggests that electron transfer to the catalytic nitrogenase center was an ancestral function of the nitrogen-fixing process. This family encompasses numerous paralogs, including those involved in bacteriochlorophyll biosynthesis in photosynthetic bacteria, sulfur metabolism mediated by cysteine desulfurases, and coenzyme F430 biosynthesis in methanogenic and methanotrophic archaea (Moser and Layer 2019). Some of these paralogs, especially those linked to methanogenesis, may carry signatures of ancient origin. By contrast, mapping the origin of the molybdenum-iron protein (NifDK) domain family (c.92.2.3) onto the domain chronology reveals that this family, which belongs to the ABE Venn group, originated relatively late, at *nd* = 0.515 during Phase IV (organismal diversification), ~ 1.8 Gy ago. This relatively recent origin of the modern molybdenum-dependent nitrogenase is consistent with substitutions rate analyses suggesting nitrogenase arose 1.5–2.2 Gy ago with methanogenic origins (Boyd et al. 2011). However, it conflicts with reconciliations of nitrogenase gene trees and species chronograms, which support an earlier origin 2.7–3.1 Gy ago (Parsons et al. 2020). The discrepancy may stem from root calibration in chronogram construction with the PhyloBayes program, which equates LUCA with the origin of life.

Tracing the origins of ferredoxins showed they were evolutionarily more recent. The [2Fe–2S] ferredoxin domain family (d.15.4.2; *nd* = 0.193) and the ferredoxins of multidomain proteins (d.58.1.5; *nd* = 0.197) that are common to all superkingdoms (ABE) and universal (ABEV), respectively, and the universal short-chain ferredoxin (d.58.1.1; *nd* = 0.287) family that is typical of low potential [4Fe–4S] ferredoxins appeared during the birth of archaeal ancestors in Phase II of the timeline (Fig. 3a), about 3.0 and 2.7 Gy ago, respectively. This phase includes ABEV, ABE, BEV, and BE families. Therefore, the tandem duplication typical of clostridial ferredoxins must have occurred 250 million years ahead of the Great Oxygenation Event (GOE) of our planet and before the emergence of families specific to superkingdoms or viruses.

The origin of the tandem duplication was investigated by analyzing the loop structures of the [4Fe–4S] ferredoxin domain superfamily (Fig. 3a; Supplementary Fig. 3; Supplementary Table 2). A chronology of loop prototypes (Aziz et al. 2023) classified by ArchDB using a stringent Density Search (DS) clustering algorithm (Bonet et al. 2014) allowed to establish which of the duplicate sequences was ancestral. The ligand-binding loop prototype MCL.EH.9.3.1, identified using a more relaxed Markov clustering (MCL) algorithm, mapped to amino acid residues 30–46 of the short-chain ferredoxin from *C. pasteurianum*. The loop embedded DS.EG.1.1.12, the oldest DS prototype of the molecule (Fig. 3b), which also mapped to residues 30–36 of the ferredoxin structures of *Chromatium vinosum* (1BLU) or *Pseudomonas aeruginosa* (2FGO). The time of origin of DS.EG.1.1.12 (*nd* = 0.279) closely matched that of the SCOP d.58.1.1 family (*nd* = 0.287) (Fig. 3a), supporting the emergence of short-chain ferredoxins in their C-terminal second half. Note that the DS.EG.1.1.12 prototype is widely distributed, especially in the ADC synthase domain (d.161.1.1; *nd* = 0.279), excisionase-like (a.6.1.7; *nd* = 0.438), Mtd domain-like (b.163.1.1; *nd* = 0.481), HAL/PAL-like (a.127.1.2; *nd* = 0.321), and N-terminal domain of DNA gyrase/MutL (d.122.1.2; *nd* = 0.214) families, with enrichments at e-value < 0.01 and percentages above 4%. This contrasts with the more restricted distribution of the MCL.EH.9.3.1 prototype, which is fundamentally confined to the [4Fe–4S] ferredoxin domain superfamily (d.58.1; *nd* = 0.197–0.495). A second prototype, DS.HE.5.50.1, spanning residues 41–55, overlaps with the C-terminal region of MCL.EH.9.3.1 and adds the C47 residue that coordinates the second [4Fe–4S] cluster. This loop appeared at *nd* = 0.464 during Phase III (diversification of Bacteria), which accounts for over half of the Venn groups of the timeline. The late appearance of a C-terminal extension to the C-terminal half suggests the primordial short-chain ferredoxin fold was more compact than the extant fold, potentially resembling a maquette-like structure.

**Fig. 4:**
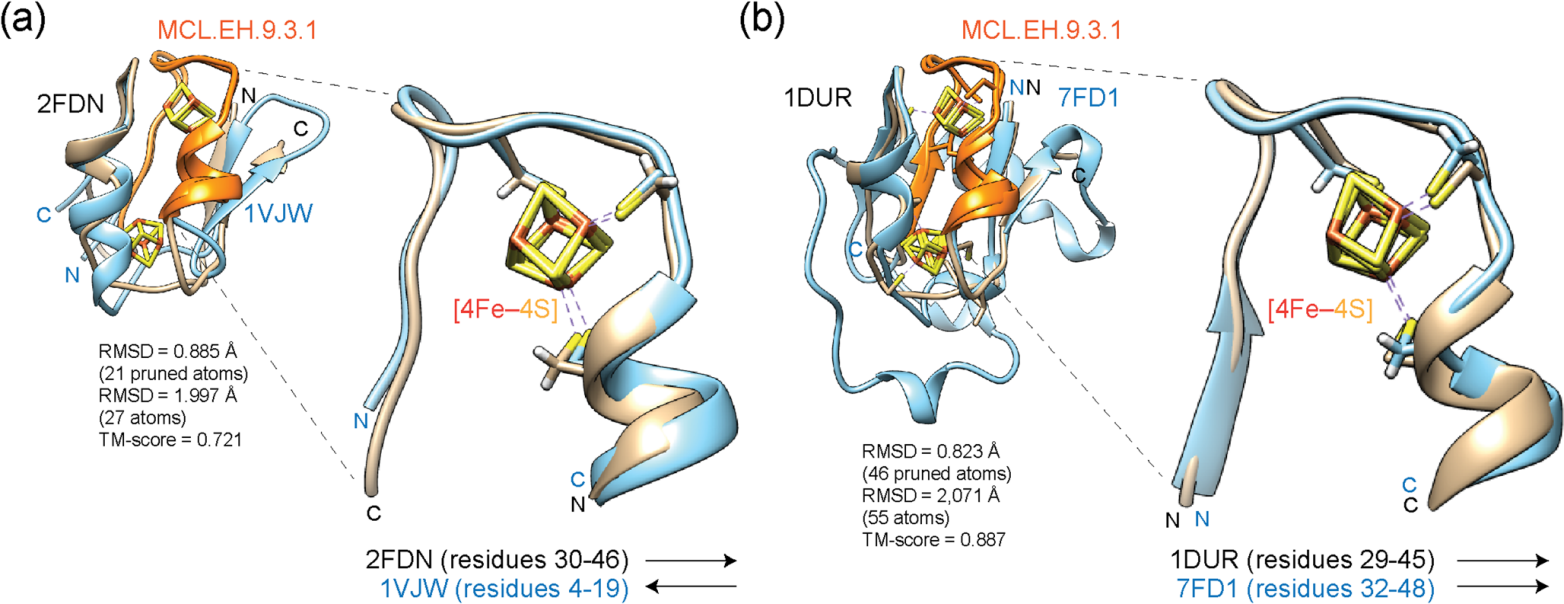
Recruitment of the MCL.EH.9.3.1 loop prototype of the short-chain ferredoxins (d.58.1.1) by single [4Fe–4S] cluster ferredoxin (d.58.1.4) (**a**) and 7Fe ferredoxin (d.58.1.2) families (**b**). The structures of short-chain ferredoxins from *Clostridium acidurici* (PDB entry 2FDN) (panel a) or from *Peptostreptococcus asaccharolyticus* (PDB entry 1DUR) (panel b) were aligned to the structures of a single [4Fe–4S] cluster ferredoxin from *Thermotoga maritima* (1VJW) and to a 7Fe ferredoxin (d.58.1.2) from *Azotobacter vinelandii* (7FD1), respectively. Their aligned MCL.EH.9.3.1 loop prototypes were excised and rendered separately. Both N- and C-termini are labeled in the models. The MCL.EH.9.3.1 loop is colored orange in the complete alignments. Arrows indicate the N- to C-terminal direction of the MCL.EH.9.3.1 chains in excised alignments

Mapping loop prototypes to domains of [4Fe–4S] ferredoxins revealed additional episodes of recruitment and diversification unfolding for the most part during a 1 Gy interval of Phase IV (organismal diversification) (Fig. 3a). The single [4Fe–4S] cluster ferredoxin (d.58.1.4), the 7Fe ferredoxin (d.58.1.2) and the archaeal ferredoxin (d.58.1.3) families originated at *nd* values of 0.463, 0.494 and 0.554, respectively. This order of appearance supports the model of [4Fe–4S] ferredoxin evolution proposed by Meyer (2008), in which a more primordial short-chain ferredoxin structure gives rise to the three ferredoxin families through cluster loss, loop creation, and C- and N-terminal extensions, now further refined with a temporal framework (Fig. 3c). During this period, emerging structures underwent additional diversification through processes of cooption and growth, some of which were captured by loop-domain mappings (Fig. 3a). A notable example is the cooption of MCL.EH.9.3.1 and its embedded DS.EG.1.1.12 prototype by single [4Fe–4S] cluster ferredoxins and 7Fe ferredoxins. Unlike short-chain ferredoxins, MCL.EH.9.3.1 was located within the first 20 residues of the N-terminal region of single [4Fe–4S] cluster ferredoxins and interacted directly with the single [4Fe–4S] cluster of the molecule. Structural alignments of the two domain families revealed a head-to-tail arrangement that placed the MCL.EH.9.3.1 loops in different halves of the two domain structures (Fig. 4a). This observation strengthens an origin of the ferredoxin ancestor in the C-terminal half of the short-chain ferredoxins by suggesting the C-terminal half of the short-chain ferredoxin precursor gave rise to the structure of single [4Fe–4S] cluster ferredoxins. In sharp contrast, the symmetrical inversion was absent in alignments between short-chain ferredoxins and 7Fe ferredoxins (Fig. 4b). The aligned structures showed both MCL.EH.9.3.1 loops were present in the second half of the two domain structures. Instead, 7Fe ferredoxins diversified by recruiting loops DS.GH.1.2.10 (*nd* = 0.494) and later DS.EG.0.1.1 (*nd* = 0.747) to help form a C-terminal extension of their structures (Fig. 3a). Finally, the emergence of archaeal ferredoxins (d.58.1.3), which do not have the MCL.EH.9.3.1 loop prototype, appears considerably more convoluted and possibly contrary to an origin in short-chain ferredoxins (Meyer 2008). The modular prototype DS.EH.8.10.1 (*nd* = 0.554), which aligns well to MCL.EH.9.3.1 of short-chain ferredoxins, was present in the N-terminal half of the archaeal molecule (Supplementary Fig. 4). The prototype was probably recruited from older multidomain formate dehydrogenase (1KQF) and Fe-only hydrogenase (1FEH) enzymes, which harbor several ferredoxin-like domain structures. For example, the Fe-only hydrogenase enzyme is a large enzyme with three domains, an ‘N-terminal domain’ holding the [2Fe–2S] ferredoxin domain (d.15.4.2; *nd* = 0.193) structure, a ‘second domain’ holding the ferredoxin domain from multidomain protein (d.58.1.5; *nd* = 0.197) structure, and a ‘catalytic domain’ holding the Fe-only hydrogenase (c.96.1.1; *nd* = 0.527) structure. An alignment of the second domain against either a short-chain ferredoxin or the archaeal ferredoxin showed the second domain embedded the DS.EH.8.10.1 prototype of archaeal ferredoxins in its N-terminal half (Supplementary Fig. 5). Both alignments confirmed all three structures collectively hold the same fold (TM-scores of 0.731 and 0.615, respectively). These results support the N-terminal loop configuration of the derived archaeal ferredoxins was probably drawn from more ancient ferredoxin structures in multidomain proteins, which appeared 3 Gy ago in the timeline. These results showcase the complexity of loop recruitments and symmetry in ferredoxin evolution.

In summary, the history of protein domains and loops shows that the relatively simple structure of primordial short-chain ferredoxins was first expanded by a tandem duplication and was subsequently refined by gradual episodes of recruitment, growth, loss and structural diversification. At the center of this history were the MCL.EH.9.3.1 and DS.EH.8.10.1 loop prototypes, with origins in the C-terminal and N-terminal halves, respectively.

## A Final Note on Margaret Dayhoff’s Visionary Insight

Eck and Dayhoff’s seminal work laid the groundwork for advancing sequence alignment and understanding evolutionary conservation. Their conviction that proteins should be viewed as ‘living relics’ or ‘documents of evolutionary history’ was revolutionary. Their identification of building blocks prompted the search for ‘prior molecular states’ capable of persisting over deep evolutionary time as historical remnants or as molecular ‘modules’ (*sensu* Simon 1962), often through cooption into new functional contexts (Caetano-Anollés et al. 2024). Whether such processes of innovation involve duplication, fusion, or other mechanisms of protein diversification remains an active area of study (Muthahari et al. 2025).

Margaret Dayhoff’s visionary insights into molecular evolution laid the foundation for many aspects of modern bioinformatics (McNeill 2019). Her pioneering use of computational tools to study protein sequences helped shape the nascent field of computational biology. Beyond the ferredoxin duplication model, Dayhoff is perhaps best known for creating the first protein database and the ‘Dayhoff matrix’ of amino acid substitutions (Chang et al. 1965), both of which remain cornerstones of sequence and structure analyses. As recalled in historical accounts (McNeill 2019), she was a trailblazer who brought modern computing to the service of biology at a time when such efforts were unprecedented. On her 100th birthday, revisiting her work celebrates a legacy that continues to guide and inspire bioinformaticians and molecular evolutionary biologists worldwide, especially women pursuing careers in these fields.

## Supplementary Information

Below is the link to the electronic supplementary material.

Supplementary Material 1

Supplementary Material 2

Supplementary Material 3

Supplementary Material 4

Supplementary Material 5

Supplementary Material 6

Supplementary Material 7
