# Supplementary material for: Revisiting Eck and Dayhoff’s Building Block Model of Ferredoxin Evolution on Dayhoff’s 100th Birthday
Source: J Mol Evol. 2025 Nov 6;94(1):52–61. doi: 10.1007/s00239-025-10283-3 (PMC12920312; doi:10.1007/s00239-025-10283-3)
Supplement: Supplementary file 1 — Supplementary Material 1 [file 239_2025_10283_MOESM1_ESM.pdf]

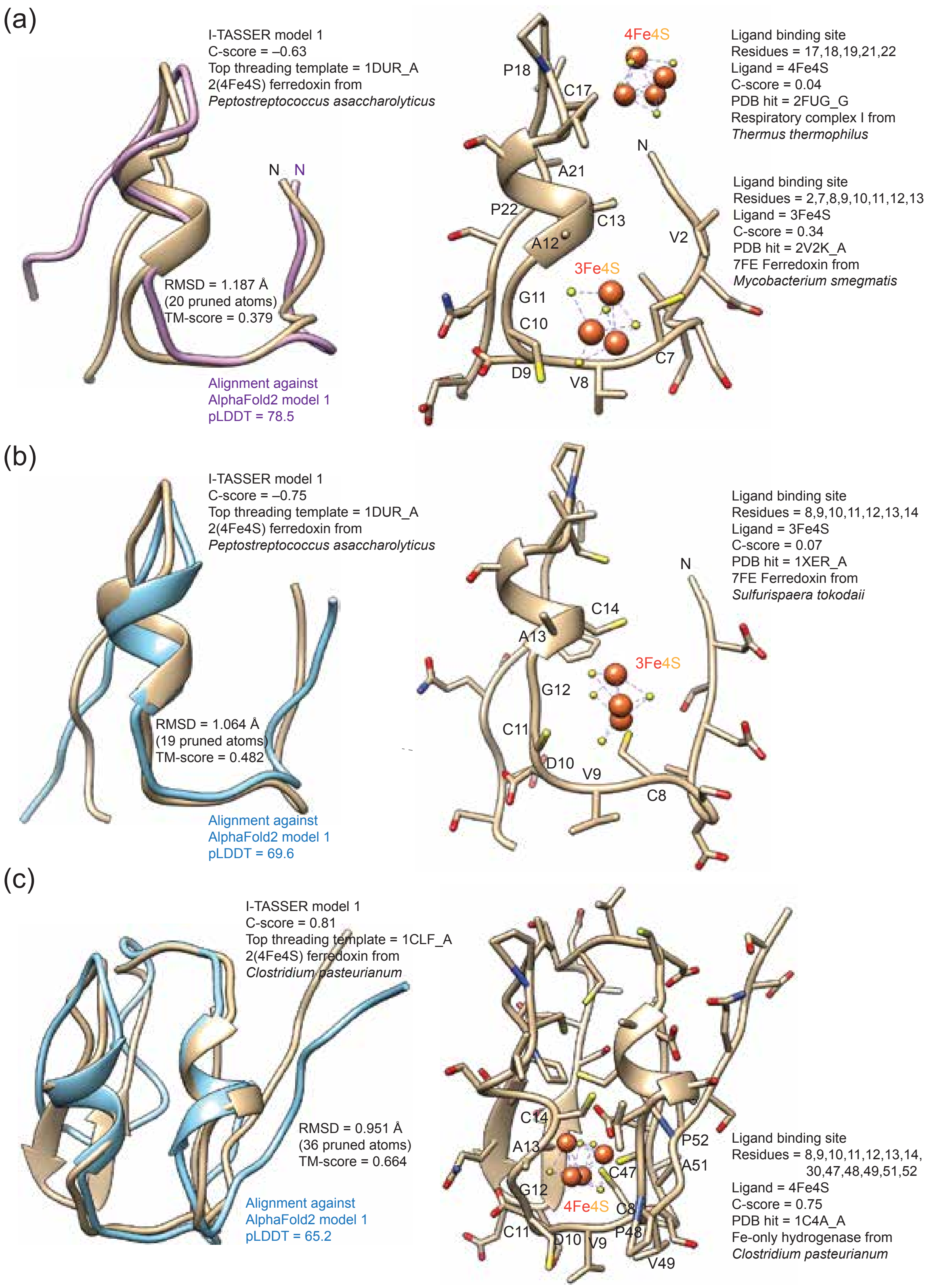

Supplementary Fig. 1 I-TASSER benchmarking of the AlphaFold2 models for the retrodicted (row 5 of Fig. 1) sequence (**a**) and the predicted sequences preceding (row 3 of Fig. 3)(**b**) or following (row4 of Fig. 3)(**c**) the tandem duplication proposed by Eck and Dayhoff. Structural alignments (left) describe superposition of atomic structures of the AlphaFold2 (blue backbone) and I-TASSER (tan backbone) models. The I-TASSER atomic models (right) describe predicted ligand binding sites and ligands for the I-TASSER models using the COFACTOR and COACH programs.
