# Supplementary material for: Revisiting Eck and Dayhoff’s Building Block Model of Ferredoxin Evolution on Dayhoff’s 100th Birthday
Source: J Mol Evol. 2025 Nov 6;94(1):52–61. doi: 10.1007/s00239-025-10283-3 (PMC12920312; doi:10.1007/s00239-025-10283-3)
Supplement: Supplementary file 2 — Supplementary Material 2 [file 239_2025_10283_MOESM2_ESM.pdf]

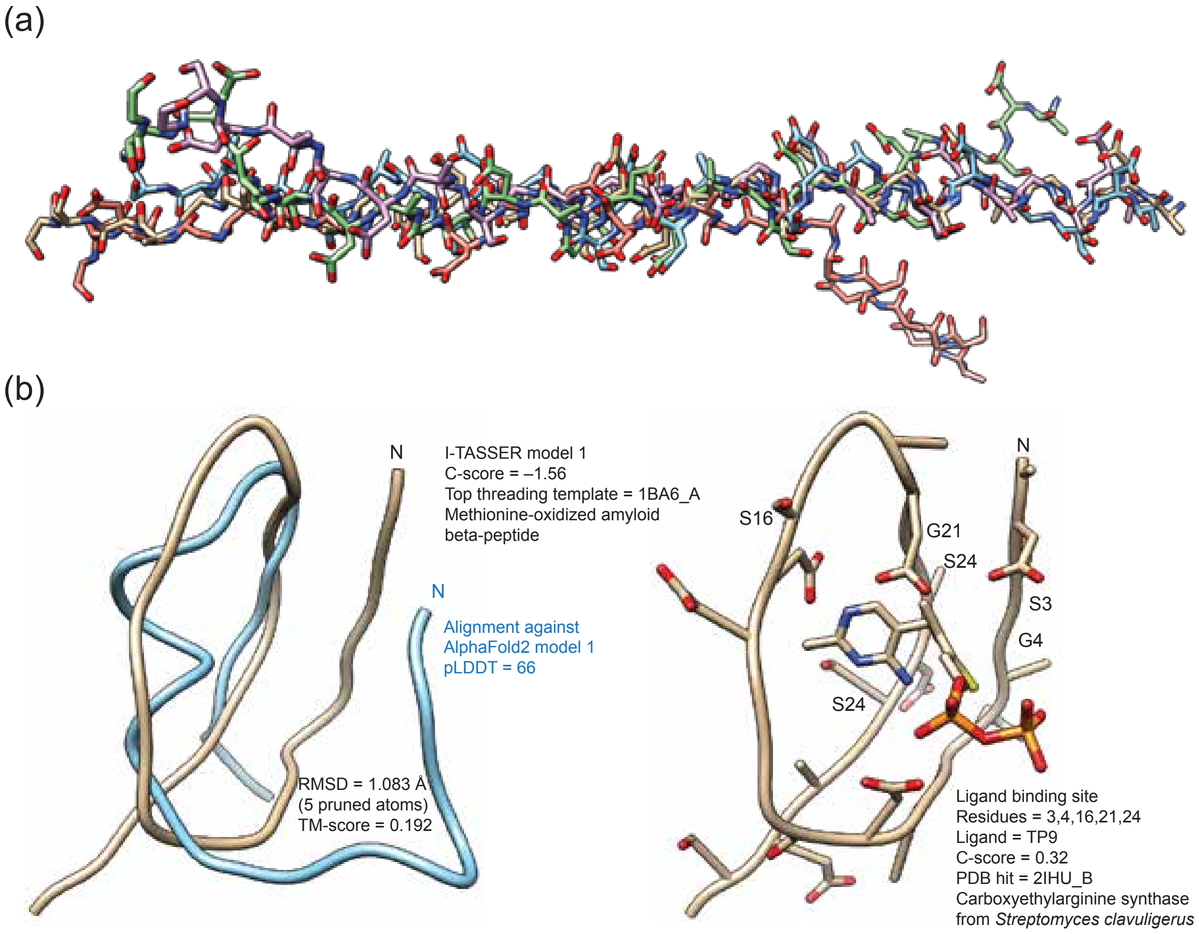

Supplementary Fig. 2 AlphaFold2 and I-TASSER models of the retrodicted sequence of row 6 of Fig. 1 (equal to predicted sequence of row 3 of Fig. 2) of the Eck and Dayhoff (1966) study. (**a**) Structural alignment of the top five AlphaFold2 ranked models show poorly aligned coil structures. (**b**) Structural alignment of the top ranked I-TASSER threaded structure of the retrodicted/predicted sequence (tan backbone) to the AlphaFold2 retrodicted structure of the retrodicted sequence of row 5 of Fig. 1 of Eck and Dayhoff (1966) (left) and the I-TASSER prediction of the best ligand binding site and ligand using the COFACTOR and COACH programs (right) .
