# Supplementary material for: Revisiting Eck and Dayhoff’s Building Block Model of Ferredoxin Evolution on Dayhoff’s 100th Birthday
Source: J Mol Evol. 2025 Nov 6;94(1):52–61. doi: 10.1007/s00239-025-10283-3 (PMC12920312; doi:10.1007/s00239-025-10283-3)
Supplement: Supplementary file 3 — Supplementary Material 3 [file 239_2025_10283_MOESM3_ESM.pdf]

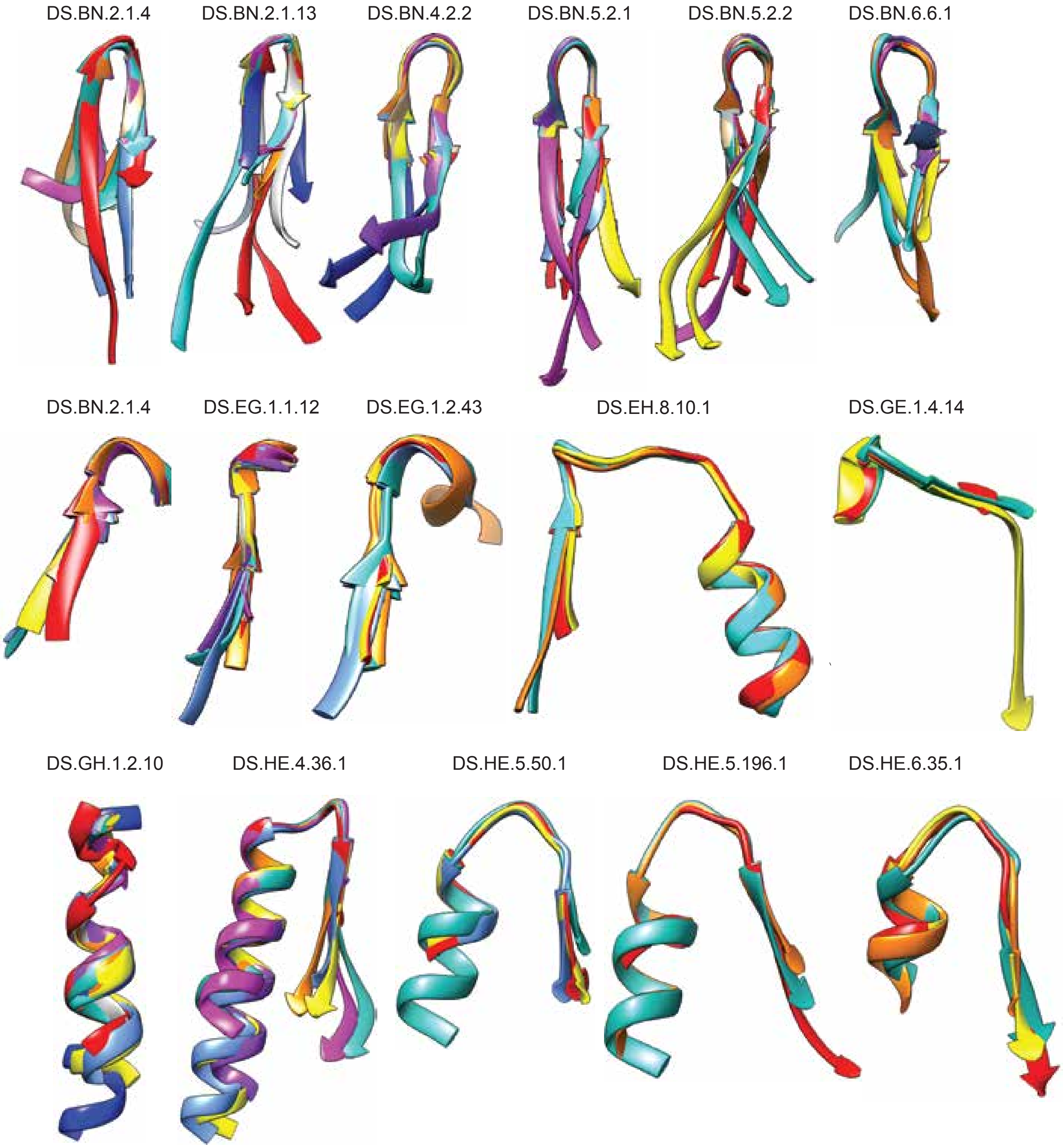

Supplementary Fig. 3 Loop prototypes of the [4Fe–4S] ferredoxin superfamily cataloged by the Density Search (DS) clustering algorithm of ArchDB.
