# Supplementary material for: Revisiting Eck and Dayhoff’s Building Block Model of Ferredoxin Evolution on Dayhoff’s 100th Birthday
Source: J Mol Evol. 2025 Nov 6;94(1):52–61. doi: 10.1007/s00239-025-10283-3 (PMC12920312; doi:10.1007/s00239-025-10283-3)
Supplement: Supplementary file 4 — Supplementary Material 4 [file 239_2025_10283_MOESM4_ESM.pdf]

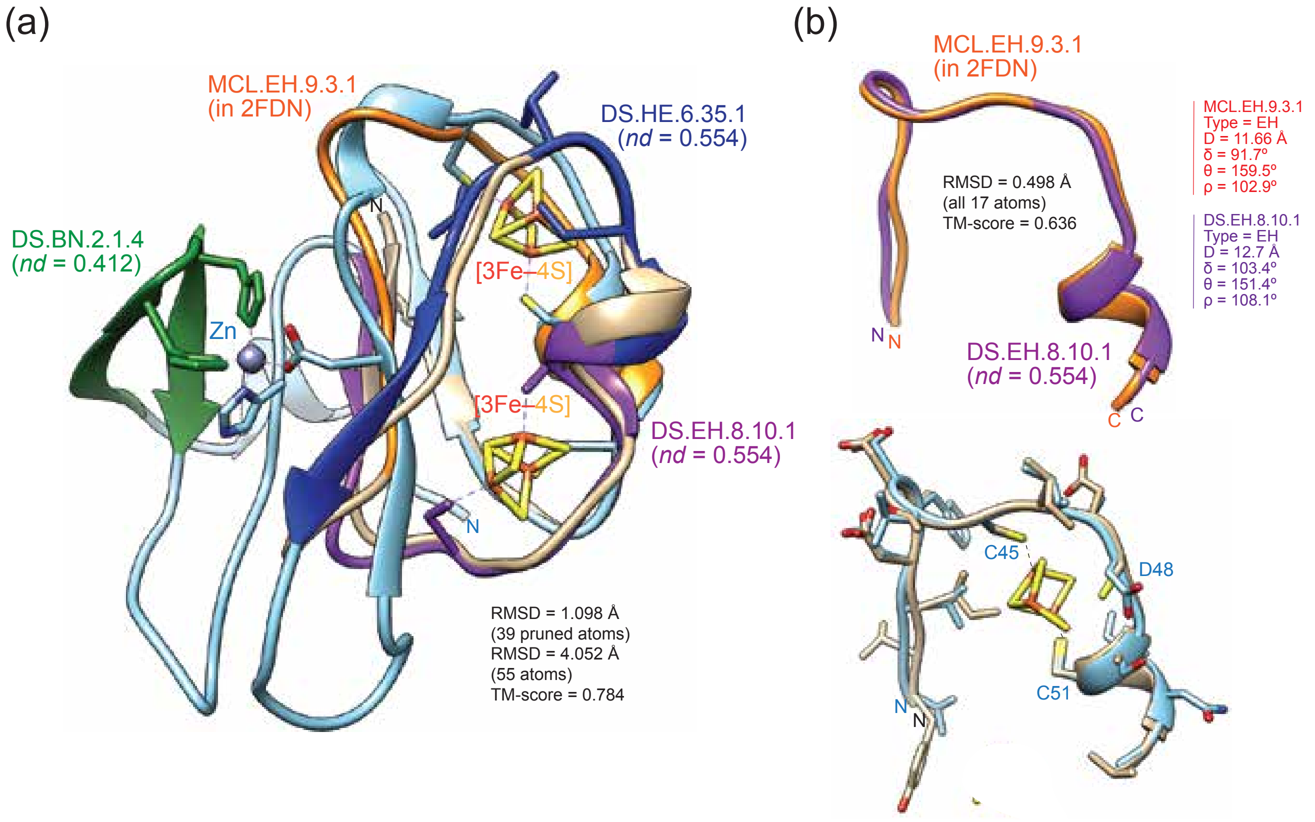

Supplementary Fig. 4 Origin of archaeal ferredoxins. (**a**) Tracing loop prototypes in an alignment of a short-chain ferredoxin (d.58.1.1) from *Clostridium acidurici* (PDB entry 2FDN colored in tan) to an archaeal ferredoxin (d.58.1.3) from *Sulfolobus* sp. (1XER colored in light blue). The MCL.EH.9.3.1 loop prototype of the reference short chain ferredoxin that is colored in orange is absent in the archaeal ferredoxin. Instead, two new loop prototypes colored in blue and purple (indexed with times of origin, *nd*) populate the N-terminal half of the archaeal molecule and coordinate with the [3Fe–4S] clusters. An extra loop prototype colored in green coordinates with a Zn ion. The region that aligns with MCL.EH.9.3.1 does not harbor a classified prototype. (**b**) A structural alignment between MCL.EH.9.3.1 (orange) and DS.EH.8.10.1 (purple) shows an overlap of the backbones (top) suggesting an evolutionary recruitment from the C-terminal half of short chain ferrredoxins to the N-terminal half of archaeal ferredoxins. The bottom depiction shows atom–bond details and coordination with the [3Fe–4S] cluster of the archaeal molecule.
