# Supplementary material for: Revisiting Eck and Dayhoff’s Building Block Model of Ferredoxin Evolution on Dayhoff’s 100th Birthday
Source: J Mol Evol. 2025 Nov 6;94(1):52–61. doi: 10.1007/s00239-025-10283-3 (PMC12920312; doi:10.1007/s00239-025-10283-3)
Supplement: Supplementary file 5 — Supplementary Material 5 [file 239_2025_10283_MOESM5_ESM.pdf]

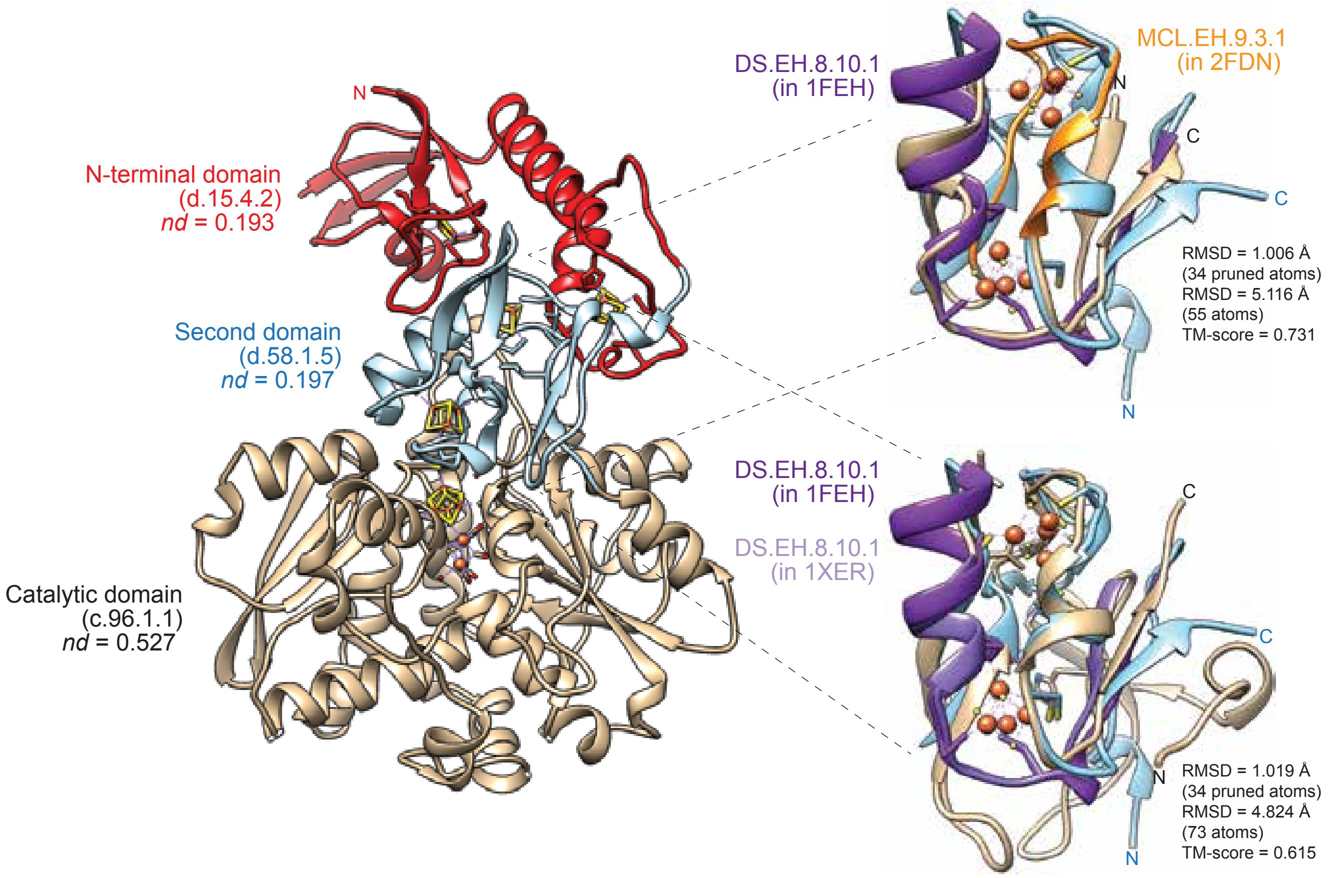

Supplementary Fig. 5 Tracing the origin of archaeal ferredoxins to Fe-only hydrogenase enzymes. A Fe-only hydrogenase from *Clostridium pasteurianum* (PDB entry 1FEH) is shown in the left with its three domains. Excising the 'second domain' (d.58.1.5) from the multidomain enzyme and aligning it to either a short-chain ferredoxin (d.58.1.1) from *Clostridium acidurici* (PDB entry 2FDN colored in tan; top right) or to an archaeal ferredoxin (d.58.1.3) from *Sulfolobus* sp. (1XER in tan; bottom right) shows that the second domain lacks the MCL.EH.9.3.1 loop prototype of the C-terminal half of the short-chain ferredoxin (orange) but instead shares the DS.EH.8.10.1 prototype of the N-terminal half of the archaeal ferredoxin (purple and light purple).
